# CD14-Positive Cells Support Osteoblast Viability and Mineralization Without Altering Proinflammatory Cytokine Responsiveness

**DOI:** 10.3390/biology15161420

**Published:** 2026-08-18

**Authors:** Juliana F. Bousch, Jannes Klenzendorf, Christoph V. Suschek, Carl Neuerburg, Christoph Beyersdorf

**Affiliations:** Department for Orthopedics and Trauma Surgery, Medical Faculty, University Hospital Düsseldorf, Heinrich Heine University Düsseldorf, 40225 Düsseldorf, Germany; julianafranziska.bousch@med.uni-duesseldorf.de (J.F.B.); jannes.klenzendorf@hhu.de (J.K.); christoph.suschek@med.uni-duesseldorf.de (C.V.S.); carl.neuerburg@med.uni-duesseldorf.de (C.N.)

**Keywords:** osteoblast culture, macrophages, CD14, cytokines

## Abstract

Bone-resident macrophages are increasingly recognized as regulators of bone metabolism, yet their contribution to primary human osteoblast cultures remains poorly understood. We investigated the functional relevance of CD14-positive cells in primary human osteoblast cultures obtained using two isolation methods. CD14-positive cells were depleted by magnetic-activated cell sorting, and non-depleted and depleted cultures were compared regarding cell viability, matrix mineralization, osteogenic and macrophage-associated marker expression, and responses to IL-1β, IL-6, and TNF-α. CD14 depletion reduced cell viability under growth conditions and significantly impaired matrix mineralization in cultures obtained with both isolation protocols. Depletion markedly decreased CD14 and other monocyte/macrophage-associated markers, while the osteogenic cell population was largely preserved, although marker-specific changes suggested altered osteoblast maturation. Despite reduced basal mineralization, IL-1β and TNF-α significantly enhanced mineralization in both non-depleted and CD14-depleted cultures, whereas IL-6 had no significant effect. These findings indicate that CD14-positive cells support basal osteoblast viability, maturation, and mineralization but are not required for the mineralization-promoting effects of IL-1β and TNF-α. Primary human osteoblast cultures should therefore be considered multicellular systems in which macrophage-like cells contribute substantially to basal osteogenic function.

## Simple Summary

Bones are constantly renewed by cells that build new bone and cells that break down old bone. This balance can be disturbed in ageing and joint disease, leading to weaker bones and impaired repair. While bone-forming cells are often studied on their own, real bone tissue also contains immune cells that may influence how well bone is formed. This study examined whether a specific group of immune-like cells, called CD14-positive cells, helps human bone-forming cells survive and produce mineralized bone-like tissue in the laboratory. We removed these cells from primary human bone-forming cell cultures and compared the results with untreated cultures. Removing CD14-positive cells reduced cell survival and clearly weakened the formation of mineralized tissue, although most bone-forming cells remained present. The study also tested inflammatory messenger proteins that are released during injury or disease. Two of these proteins still increased mineralized tissue formation even after CD14-positive cells had been removed, suggesting that their effects do not depend mainly on these cells. Overall, the findings show that human bone-forming cell cultures are mixed cell systems and that CD14-positive immune-like cells support normal bone-forming activity. This knowledge may help improve future laboratory models of bone disease and repair.

## 1. Introduction

Bone is a highly dynamic tissue that is continuously remodeled throughout life. Approximately 10% of the skeletal mass is renewed each year through the tightly coordinated activities of bone-forming osteoblasts and bone-resorbing osteoclasts [1]. With advancing age, this homeostatic balance becomes increasingly disrupted, as chronic low-grade inflammation enhances bone resorption, impairs bone formation, and thereby contributes to progressive skeletal degeneration.

Osteoblasts arise from mesenchymal progenitor cells and progressively differentiate into mature matrix-producing cells, some of which ultimately become embedded within the mineralized bone matrix as osteocytes [2]. In a previous study, we systematically investigated the effects of pro-inflammatory cytokines on osteogenic differentiation, proliferation, and metabolic activity of primary human osteoblasts. However, accumulating evidence from the field of osteoimmunology indicates that bone metabolism is not governed by osteoblasts and osteoclasts alone, but is critically shaped by immune cells within the bone microenvironment, particularly macrophages [3,4].

Macrophages display remarkable functional plasticity in response to local environmental cues. Classically activated, pro-inflammatory M1-like macrophages are generally associated with inflammatory tissue degradation and enhanced bone resorption, whereas alternatively activated, M2-like macrophages have been linked to tissue repair, bone formation, and the release of osteogenic growth factors and anti-inflammatory cytokines [5,6]. Consequently, macrophage modulation has emerged as a promising strategy to influence bone regeneration and inflammatory bone loss.

Of particular interest are bone-resident macrophages, commonly referred to as osteomacs [7]. These cells are located in close proximity to osteoblasts and have been described as forming characteristic canopy-like structures in which osteomacs extend over and contact mature osteoblasts along active endosteal bone-forming surfaces. In mice, such structures were reported to cover more than 75% of mature osteoblasts [7]. Beyond their role in bone formation, osteomacs may also contribute to the resorption phase through phagocytic activity and local tissue remodeling [8]. In murine fracture models, osteomacs have been shown to be important for bone repair [9], supporting the concept that they exert pleiotropic functions depending on their phenotype, activation state, and microenvironment. Nevertheless, their precise role in healthy and diseased human bone remains insufficiently understood.

Importantly, macrophages are co-isolated in osteoblast cultures. In murine calvarial preparations, osteomacs accounted for approximately 16% of the isolated osteal cell population and were broadly distributed along endosteal and periosteal bone surfaces [7]. This raises the possibility that cellular functions previously attributed exclusively to osteoblasts may, at least in part, be mediated by macrophages or by osteoblast–macrophage interactions. Chang and colleagues demonstrated that murine macrophage-depleted osteoblast cultures exhibited increased expression of the osteogenic markers *ALPL* and *COL1A1*, but reduced expression of osteocalcin, suggesting that macrophages may contribute specifically to osteoblast maturation and mineralization rather than to early differentiation or proliferation [7].

To date, however, little is known about the presence and functional relevance of macrophage-like CD14-positive cells in human primary osteoblast cultures. We recently succeeded in isolating human osteomacs from femoral heads for the first time [10]. These advances provide the basis for disease-specific analyses of human bone-resident macrophage function.

Previous murine studies have demonstrated that macrophages are essential for osteoblast function in vitro and in vivo, including in models of bone healing [11]. Moreover, macrophage polarization appears to be critical, with M2-like phenotypes being particularly supportive of osteogenesis and tissue repair [12].

Because isolation protocols can influence the composition and functional behavior of primary bone-derived cultures, we additionally considered the impact of two osteoblast isolation approaches. The first protocol (hOB-I) used limited washing and collagenase type IV digestion, whereas the second protocol (hOB-II) was developed by our group in the context of isolating tissue-resident osteal macrophages from human bone. In this protocol, repeated washing steps were introduced to more extensively remove bone marrow and thereby reduce co-isolation of bone marrow-derived macrophages (BMDMs), and collagenase type II was used instead of collagenase type IV based on reports suggesting improved suitability for bone tissue digestion [13]. This comparison allowed us to assess whether the modified isolation procedure affects osteoblast viability and mineralization and whether these effects are influenced by subsequent CD14 depletion. The hOB-II protocol was designed to reduce the co-isolation of bone marrow-derived cells through repeated washing steps before enzymatic digestion. Consequently, the remaining CD14-positive population may be relatively enriched for bone-associated, osteomac-like macrophages. However, the protocol does not selectively isolate osteomacs, and residual bone marrow-derived macrophages cannot be excluded. Accordingly, comparisons between non-depleted and CD14-depleted cultures assess the contribution of the overall CD14-positive compartment rather than permitting lineage-specific attribution to osteomacs.

The aim of the present study was to investigate the functional relevance of CD14-positive cells in primary human osteoblast cultures. Specifically, we compared CD14-depleted and non-depleted osteoblast cultures with regard to the expression of osteogenic and macrophage-associated markers, proliferation, mineralization capacity, and the response to pro-inflammatory cytokines. By clarifying the contribution of CD14-positive cells to osteoblast culture behavior, this study provides a foundation for future investigations into osteoblast–macrophage interactions in disease-relevant human bone models.

## 2. Materials and Methods

### 2.1. Materials

Unless otherwise stated, all chemicals, antibodies, and assay kits were sourced from Sigma-Aldrich Chemie GmbH (Munich, Germany).

### 2.2. Bone Material and Ethics Approval

The osteoblast cells included in this study were isolated from patients who underwent joint replacement surgery at the Department of Orthopedics and Trauma Surgery at the University Hospital Düsseldorf, Germany, due to osteoarthritis or a fracture of the hip. The use of these cultures was approved by the Local Research Ethics Committee of Heinrich Heine University Düsseldorf (Study No. 2016-2768). The patients were informed and provided written consent.

### 2.3. Isolation and Culture of Primary Human Osteoblasts

Primary human osteoblasts were isolated using two different protocols. Cells obtained using the first isolation method are hereafter referred to as hOB-I. Details of this protocol have been described previously [14,15]. The main differences compared with the second isolation method, hereafter referred to as hOB-II, were the inclusion of only a single washing step prior to collagenase digestion and incubation of the tissue with collagenase type IV (2.5 mg/mL; Gibco by Life TechnologiesTM, Carlsbad, CA, USA) for 2.5 h at 37 °C. For the isolation of hOB-II, the trabecular bone tissue was washed more thoroughly and subsequently subjected to sequential enzymatic digestion with collagenase type II (2.5 mg/mL) and dispase II (2.5 mg/mL). Digestion was performed in five consecutive 15 min intervals over a total period of 75 min, resulting in five fractions. Cells obtained from fractions II-V were pooled. A detailed description of this isolation protocol has also been published previously [11]. For cell growth, hOBs were cultured in Dulbecco’s Modified Eagle Medium (DMEM; Gibco by Life Technologies^TM^, Carlsbad, CA, USA) with 1% Penicillin/Streptomycin and 10% FCS (PAN-Biotech, Aidenbach, Germany) at 37 °C with 5% CO_2_ (growth medium; GM). For the induction of osteogenesis, cells were incubated in GM supplemented with 50 µM L-ascorbic acid 2-phosphate, 10 mM β-glycerophosphate disodium salt hydrate, and 500 nM dexamethasone (osteogenesis induction medium; OIM).

### 2.4. Depletion of CD14-Positive Cells by MACS^®^

Magnetic-activated cell sorting (MACS^®^; Miltenyi Biotec, Bergisch Gladbach, Germany) was used to deplete CD14-positive cells from hOB cultures. Briefly, hOBs were resuspended in phosphate-buffered saline (PBS) supplemented with 0.5% BSA and 2 mM EDTA (MACS buffer) and incubated with human CD14 MicroBeads for 15 min at 4 °C. The cell suspension was then applied to an MS Column, which was washed three times with 500 µL of MACS buffer. The flow-through containing the CD14-negative cell fraction was collected, and the cells were cultured at 37 °C before being used in subsequent experiments. The procedure was performed according to the manufacturer’s protocol.

### 2.5. Cell Viability Assay

Cell viability was assessed using a dimethylthiazolyl blue tetrazolium bromide (MTT) assay. In 96-well plates, 1 × 10^3^ cells per well were seeded and incubated with MTT in growth medium for 2 h at 37 °C. The resulting formazan crystals were subsequently dissolved by incubation with dimethyl sulfoxide (DMSO) for 10 min. Absorbance was measured photometrically at 550 nm [16].

### 2.6. Alizarin Red S Staining

To assess osteoblastic differentiation, alizarin red staining was used to quantify matrix mineralization. hOBs were seeded in 48-well plates at a density of 2.5 × 10^4^ cells per well. On days 1, 14, and 21, the cells were fixed with 4% formaldehyde for 15 min and incubated with a 2% alizarin red S monosodium salt staining solution in ddH2O for 20 min at 37 °C. After several washing steps with ddH_2_O, the bound dye was solubilized by incubation with 10% (1 hexadecyl) pyridinium chloride monohydrate for 1 h. Absorbance was measured photometrically at 660 nm [17]. For each donor, mineralization values measured on days 14 and 21 were normalized to the corresponding day 1 value of the same donor. Technical replicates were averaged before donor-specific normalization and statistical analysis. Recombinant human IL-1β, IL-6, and TNF-α were purchased from PeproTech (Thermo Fisher Scientific GmbH, Karlsruhe, Germany). Based on our previous concentration–response experiments, IL-1β and TNF-α were applied at 250 U/mL, as their effects increased in a concentration-dependent manner, whereas IL-6 was applied at 12.5 U/mL as a representative effective concentration that produced a consistent effect on primary human osteoblasts [14].

### 2.7. Real Time Quantitative PCR

For the quantification of mRNA expression of the osteogenic markers *ALPL*, *BGLAP*, *SPP1*, *RUNX2*, and *NGFR*, as well as the macrophage markers *CD14*, *PTPRC*, *CD68*, *CD86*, *CD163*, and *CD209*, cells were seeded in 6-well plates at a density of 2 × 10^5^ cells per well and cultured in growth medium for 48 h. For the isolation of total cellular RNA, cells were lysed in RLT buffer supplemented with β-mercaptoethanol at a ratio of 1:100 using the RNeasy^®^ Mini Kit (Qiagen, Hilden, Germany). RNA was subsequently purified according to the manufacturer’s instructions. For cDNA synthesis using the Omniscript^®^ RT Kit (Qiagen, Hilden, Germany), 1000 ng of total RNA was used. RT-qPCR was performed using Power SYBR^®^ Green PCR Master Mix (Thermo Fisher Scientific GmbH, Karlsruhe, Germany) in a total reaction volume of 25 µL. Transferrin receptor protein 1 (TFRC) was used as the reference gene. The primer sequences are provided in Table 1. Each sample was analyzed in technical triplicates. Relative gene expression was calculated using the 2^−ΔΔCt^ method, with normalization to TFRC and to the non-CD14-depleted hOB-II control culture.

### 2.8. Immunofluorescence Staining

Immunofluorescence staining was performed to quantify cells expressing mesenchymal/osteogenic markers (ALP, OCN, OPN, RUNX2, and CD271) or monocyte/macrophage markers (CD14, CD45, CD68, CD86, CD163, and CD209) in hOB-II cultures. Cells were seeded onto glass coverslips in 24-well plates at a density of 4 × 10^4^ cells per well and allowed to adhere for 24 h at 37 °C. Following fixation with 4% formaldehyde for 15 min, cells intended for the detection of the intracellular markers ALP, OCN, OPN, and RUNX2 were permeabilized with 0.1% Triton-X-100 for 10 min. All samples were blocked with 5% normal goat serum (NGS) for 1 h at 37 °C and incubated with the respective primary antibodies (Table 2) overnight at 4 °C. After three washing steps with PBS, the cells were incubated with the corresponding secondary antibodies (goat anti-mouse AF488 (#A-10680, Thermo Fisher Scientific GmbH, Karlsruhe, Germany) or goat anti-rabbit AF647 (#4414S, Cell Signaling Technology, Danvers, MA, USA)) for 1 h at room temperature. Following three additional washing steps with PBS, the coverslips were mounted onto microscope slides using Mowiol mounting medium containing DAPI at a dilution of 1:1000. Fluorescence images were acquired using a Keyence BZ-X800 fluorescence microscope, with four technical replicates analyzed per condition. The DAPI signals and the positive cells for each marker were counted using ImageJ (Fiji, version 1.54r).

### 2.9. Statistics

Because the analyses were based on relatively small numbers of primary human donor cell cultures, the assumption of normally distributed data could not be assessed reliably. Therefore, non-parametric statistical tests were used throughout the study. Donor characteristics, including age, sex, diagnosis, and medication, are provided in Supplementary Table S1, while Supplementary Table S2 specifies the donor cell cultures included in each analysis. Comparisons between the cell cultures of the two isolation methods (hOB-I and hOB-II), which were performed using cells from different donors, were analyzed using unpaired Mann–Whitney U tests. In contrast, comparisons between control and CD14-depleted cultures, which were derived from the same donors were analyzed using paired tests, including the Wilcoxon signed-rank test or the Friedman test followed by Dunn’s multiple comparisons test. The *n* values reported in the figure legends refer to the number of independently analyzed biological donors. The number of technical replicates per donor is additionally specified in the respective figure legends. Statistical analyses were performed using GraphPad Prism version 8 (GraphPad Software, Inc., Boston, MA, USA).

## 3. Results

**Figure 1:**
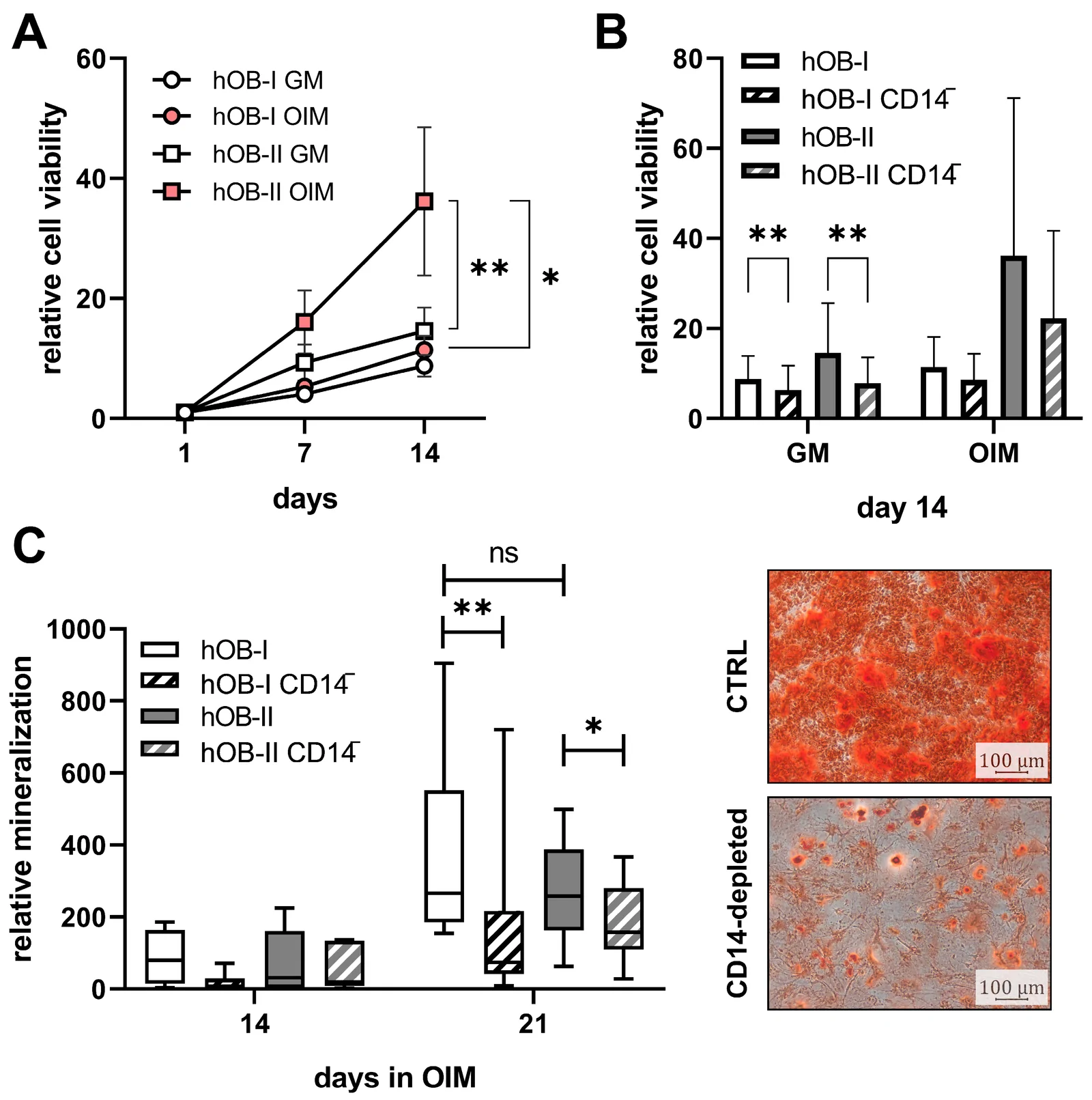
Cell viability and mineralization of osteoblasts isolated using different methods and the impact of CD14 depletion. (**A**) Cell viability of hOB-I and hOB-II cultures in GM and OIM on days 1, 7, and 14 (*n* = 8, 4 technical replicates, mean ± SEM is shown). (**B**) Cell viability of hOB-I and hOB-II CD14-depleted cultures on day 14 in GM and OIM (*n* = 8, 4 technical replicates, mean ± SD is shown), normalized on day 1 (hOB-I GM). (**C**) Mineralization of hOB-I and hOB-II CD14-depleted cultures in OIM on days 14 and 21 (hOB-I: *n* = 8; hOB-II: *n* = 7, 4 technical replicates each), normalized on day 1 of each donor. The boxplots show the median as a horizontal line, with whiskers indicating the minimum and maximum values. Wilcoxon signed-rank test was used to compare CTRL and CD14-depleted cells within a culture. Mann–Whitney U test was used to compare the hOB-I and hOB-II cultures (*p* ≤ 0.05 (*), *p* ≤ 0.01 (**)). ns = not significant.

Primary human osteoblast cultures (hOB-I and hOB-II) obtained using two isolation methods were compared in terms of cell viability under growth conditions and during osteogenic differentiation. By day 14, the hOB-II cultures in osteogenic induction medium (OIM) exhibited significantly higher cell viability than the hOB-I cultures. Additionally, the cell viability of the hOB-II cultures in OIM was significantly higher than in growth medium (GM). However, only a trend toward increased cell viability was observed in the hOB-I cultures in OIM compared to GM (Figure 1A). These results suggest that hOB-II cultures have a higher cell proliferation capacity, particularly under osteogenic conditions.

To investigate the contribution of CD14-positive cells, we compared non-depleted control cultures with CD14-depleted cultures. Under growth conditions, CD14 depletion led to a significant reduction in cell viability on day 14 in both hOB-I and hOB-II cultures. Also under osteogenic conditions, lower cell viability was observed in the CD14-depleted cultures, but not statistically significant (Figure 1B). Osteogenic differentiation of the cultures was assessed using alizarin red staining to determine mineralization. On day 21, mineralization following CD14 depletion was significantly reduced in the hOB-I and hOB-II cultures. However, there was no significant difference in mineralization between the two cultures (Figure 1C).

Although the isolation method influences cell viability under osteogenic conditions, it does not affect the cultures’ mineralization capacity. The consistent reduction in mineralization and cell viability in GM following CD14 depletion suggests that CD14-positive cells contribute to higher cell activity and consequently, the formation of a mineralized matrix in osteoblast cultures.

To characterize the heterogeneous hOB-II cell cultures further, we examined osteogenic and stromal markers, as well as markers of the monocyte and macrophage lineages, at the mRNA and protein levels.

**Figure 2:**
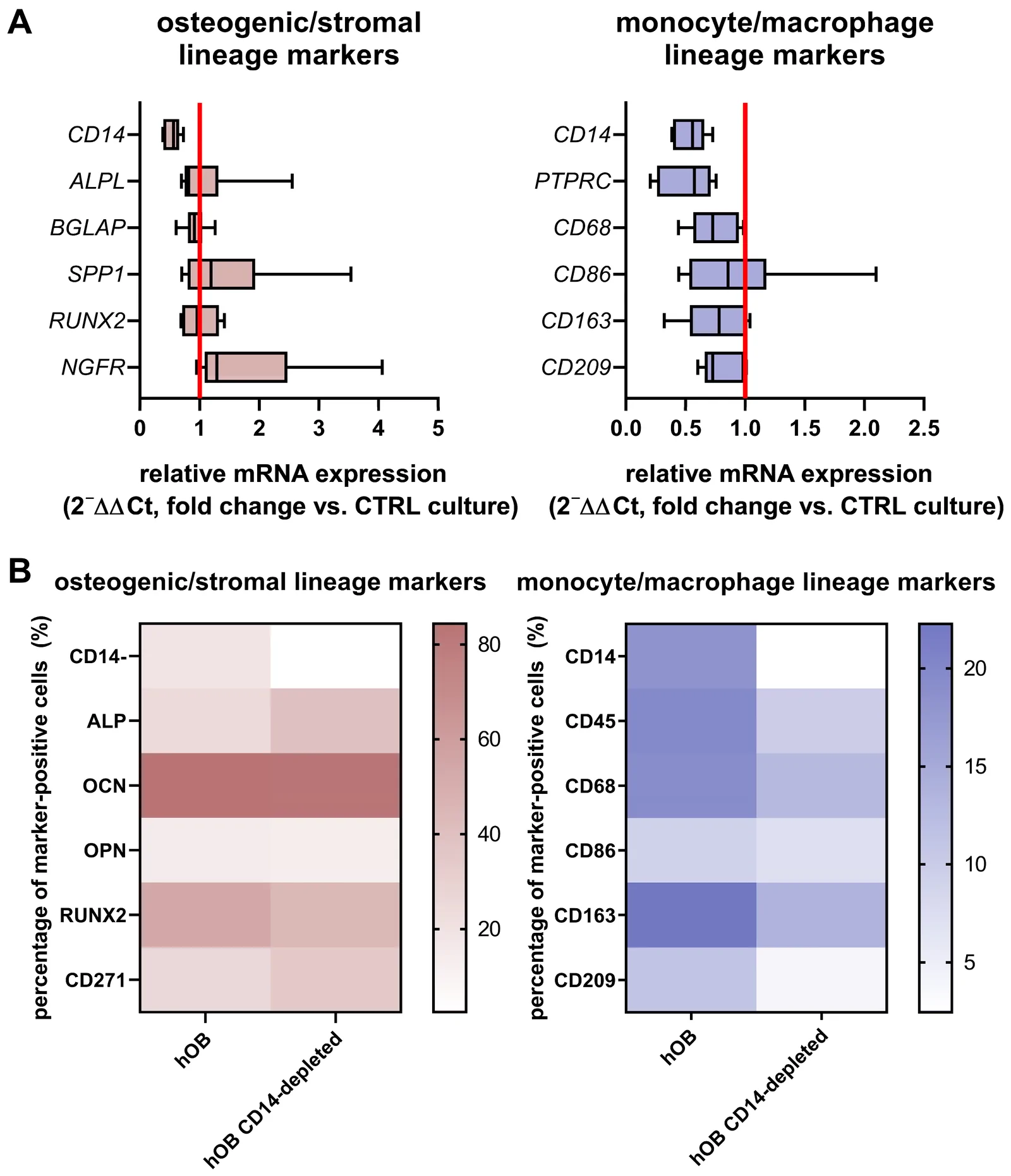
Comparison of osteogenic/stromal and monocyte/macrophage lineage markers of hOB and CD14-depleted hOB cultures. In (**A**), the mRNA expression values of osteogenic/stromal markers (red) and monocyte/macrophage markers (blue) of six individual experiments (*n* = 6, 3 technical replicates) are shown as boxplots with median values and whiskers representing minimum and maximum. The data show the fold change in the CD14-depleted culture to the control hOB-II culture (presented as the red line), analyzed by the 2^−ΔΔCt^ method. (**B**) shows the colour-coded percentage of positive cells (%) for osteogenic/stromal markers (red) and monocyte/macrophage markers (blue) in hOB-II and CD14-depleted hOB-II cultures, determined by immunofluorescence staining. The data represent the mean of three separate experiments (*n* = 3, 4 technical replicates) analyzed using ImageJ by Fiji.

Analysis of mRNA expression confirmed the reduction in CD14 following depletion (Figure 2A). The expression of the macrophage markers *PTPRC*, *CD68*, *CD86*, *CD163*, and *CD209* was also generally reduced, with median fold-change values below 1. Among the osteogenic markers, *ALPL*, *BGLAP*, and *RUNX2* showed median fold-change values close to 1, suggesting no consistent effect of CD14 depletion. *SPP1* expression moderately increased, whereas *NGFR* expression showed a more pronounced increase following depletion in all donors.

Successful depletion of CD14-positive cells was also supported by immunofluorescence analysis. Across the three donors, the mean proportion of CD14-positive cells was lower in CD14-depleted cultures than in non-depleted cultures (18.3% versus 2.5%). The proportions of CD45-, CD68-, CD86-, CD163-, and CD209-positive cells likewise showed donor-dependent decreases after CD14 depletion. Among the remaining macrophage-lineage populations, CD86-positive cells represented the largest fraction. These descriptive findings are consistent with a reduction in the macrophage-associated component of the hOB-II cultures.

The proportion of OCN-positive cells remained high in both culture conditions, with similar mean values before and after CD14 depletion (84% and 82%, respectively). ALP- and CD271-positive cell fractions tended to be higher after depletion, whereas RUNX2-positive cell fractions tended to be lower. However, the magnitude and direction of these differences varied among the three donors. Thus, the immunofluorescence data do not indicate a general loss of osteogenic cells but suggest donor-dependent, marker-specific trends following CD14 depletion. Individual donor values are shown in Supplementary Figure S1.

**Figure 3:**
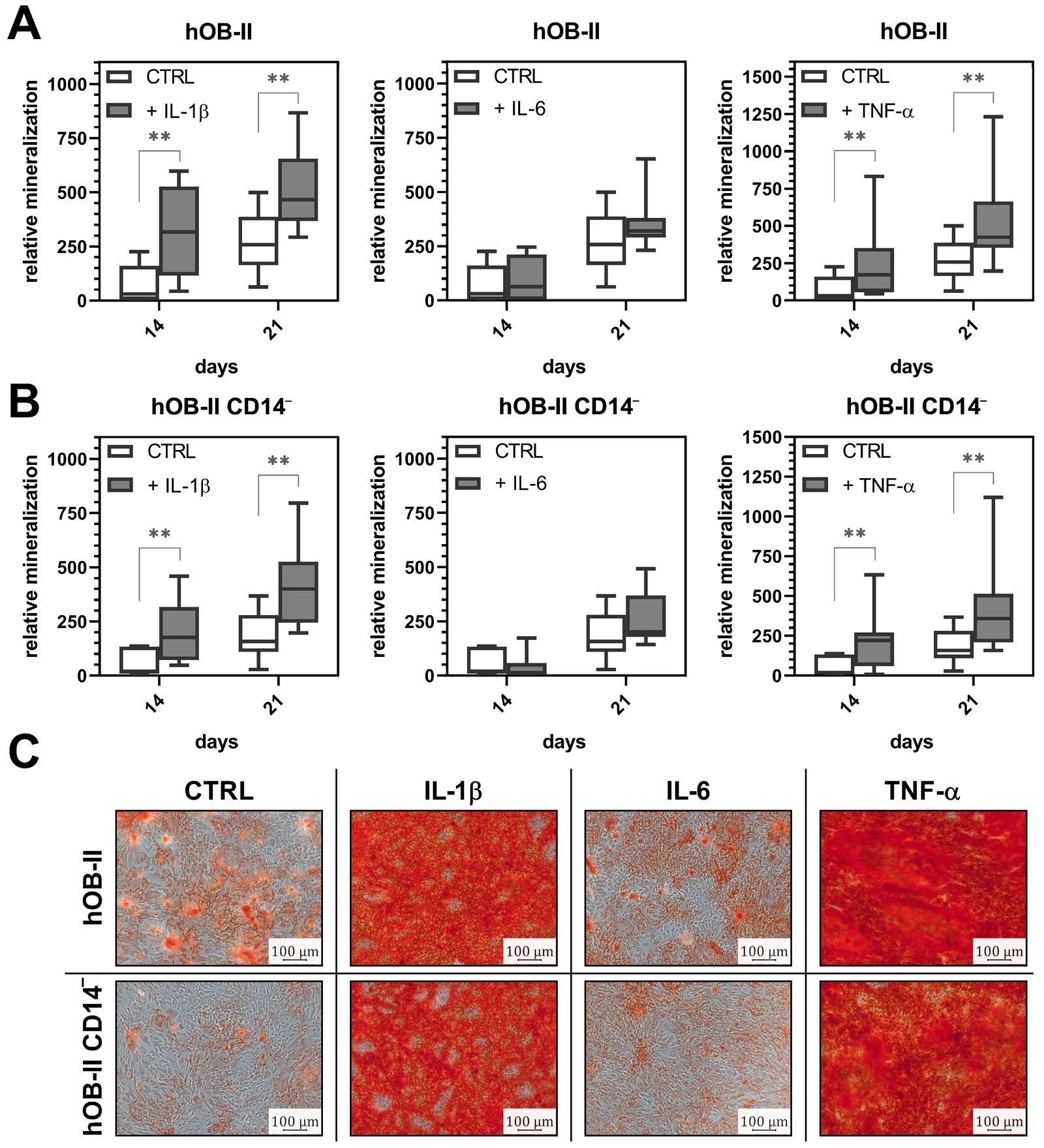
CD14 depletion reduces mineralization capacity but preserves the response to IL-1β and TNF-α in hOB-II cultures. Relative mineralization of (**A**) hOB-II and (**B**) hOB-II CD14-depleted cultures at day 14 and 21 in OIM, with and without stimulation with 250 U/mL IL-1β, 12.5 U/mL IL-6, and 250 U/mL TNF-α. Data were normalized on day 1 of each donor’s control culture and are presented as box plots showing the median with minimum-to-maximum whiskers (*n* = 7, 4 technical replicates). The statistics were calculated using the Friedman test followed by Dunn’s multiple comparisons test (*p* ≤ 0.01 (**)). (**C**) Representative microscopy example images of alizarin red staining from one donor.

The contribution of CD14-positive cells to the cytokine-induced mineralization response of hOB-II cultures was assessed by comparing control and CD14-depleted cultures following treatment with IL-1β, IL-6, or TNF-α (Figure 3). The untreated control groups correspond to those presented in Figure 1C and show the previously described lower mineralization of CD14-depleted cultures.

At days 14 and 21 of osteogenesis, IL-1β and TNF-α significantly increased mineralization compared with the respective untreated controls in both control and CD14-depleted hOB-II cultures. In contrast, IL-6 did not significantly affect mineralization at either time point. These findings suggest that the CD14-positive cell compartment supports the overall mineralization capacity of hOB-II cultures but is not required for the mineralization response to IL-1β and TNF-α.

## 4. Discussion

In the present study, we provide a first comparative analysis of primary human osteoblast cultures before and after depletion of CD14-positive cells. Our findings indicate that the CD14-positive cell fraction substantially contributes to the cellular and functional phenotype of human osteoblast cultures. In particular, osteoblast proliferation and differentiation as well as basal mineralization capacity were altered after CD14-positive cell depletion, whereas the osteoblast response to pro-inflammatory cytokine stimulation appeared to be largely preserved. These findings suggest that CD14-positive cells support basal osteoblast function and maturation but are not essential mediators of cytokine-induced mineral deposition under the present culture conditions.

The comparison of hOB-I and hOB-II indicates that methodological differences during initial cell isolation can influence subsequent culture performance. Although both protocols yielded cultures with comparable mineralization capacity as determined by alizarin red S staining, hOB-II cultures appeared to maintain higher viability over the 14-day observation period. This is particularly noteworthy because the hOB-II protocol includes more extensive washing steps and was therefore expected to reduce co-isolation of bone marrow-derived macrophages (BMDMs). Despite this anticipated reduction in BMDM contamination, hOB-II cultures performed at least as well as hOB-I cultures and showed improved long-term viability. Importantly, cultures before CD14 depletion showed higher viability and mineralization capacity than their CD14-depleted counterparts with both isolation protocols, indicating a positive effect of retaining CD14-positive cells within primary osteoblast cultures. CD14 depletion is not lineage-specific and therefore does not distinguish tissue-resident osteal macrophages from residual bone marrow-derived macrophages. Because the hOB-II protocol includes extensive washing intended to reduce retained bone marrow, its remaining CD14-positive population may be enriched for bone-associated, osteomac-like cells. However, residual bone marrow-derived macrophages cannot be excluded, and the observed effects should therefore be attributed primarily to the CD14-positive compartment rather than specifically to osteomacs. Taken together, these findings support the concept that CD14-positive cells contribute positively to basal osteoblast culture performance. In the context of the hOB-II protocol, which was designed to better preserve tissue-resident osteal macrophages while minimizing BMDM contamination, this raises the hypothesis that bone-resident macrophages may contribute to osteoblast viability and mineralization. However, the present data do not distinguish their effects from those of other CD14-positive macrophage populations. Although the mineralization assays were initiated with confluent cultures to minimize differences in initial cell density, mineralization was not normalized to endpoint cell number. Therefore, the reduced total mineralization observed after CD14 depletion cannot be attributed exclusively to an altered mineralizing function and may, at least in part, reflect differences in cell abundance or viability. Thus, the effects of isolation method and CD14 depletion should be interpreted together, as both shape the cellular composition and functional behavior of primary human osteoblast cultures.

These observations partially differ from the findings reported by Chang and colleagues, who demonstrated that macrophage depletion from murine osteoblast cultures increased *ALPL* and *COL1A1* expression while reducing osteocalcin (*BGLAP*) expression. Functionally, macrophage-depleted murine cultures showed reduced mineralization capacity [7]. Our data confirm the reduced total matrix mineralization after depletion of CD14-positive cells. At the molecular level, however, CD14-positive cell depletion in our human cultures increased *NGFR* and *SPP1* expression, whereas *ALPL*, *BGLAP* and *RUNX2* expression remained largely unchanged. In the immunofluorescence analysis, the mean proportions of CD271- and ALP-positive cells were higher after CD14 depletion, whereas RUNX2 positivity was lower and OCN positivity remained similar. However, these observations were based on three biological donors and showed donor-dependent variability. They should therefore be interpreted as descriptive, marker-specific trends rather than as evidence of a defined shift in osteoblast differentiation. Together with the reduced mineralization capacity, the findings raise the possibility that CD14 depletion affects osteoblast maturation toward an activated early-to-intermediate osteogenic phenotype while impairing or delaying terminal osteoblast maturation and matrix mineralization. However, the present marker data do not allow firm conclusions regarding the direction or stage specificity of this effect. Rather, it appears to shift the cultures toward an activated early-to-intermediate osteogenic phenotype while impairing or delaying terminal osteoblast maturation and matrix mineralization.

RUNX2 is a key transcriptional regulator of osteoblast lineage commitment and early osteoblast differentiation, while ALP is typically associated with matrix maturation and the early mineralization phase [18]. In our cultures, CD14-positive cell depletion did not substantially alter *RUNX2* or *ALPL* mRNA expression. At the protein level, the mean RUNX2-positive fraction was lower and the mean ALP- and CD271-positive fractions were higher after depletion, although these patterns varied among donors. These descriptive trends may be compatible with changes in the relative representation or state of early osteogenic and stromal cells, but they do not demonstrate an enrichment of a specific differentiation stage. Further studies with larger donor numbers and additional lineage-resolved analyses will be required to determine whether CD14 depletion reproducibly alters osteoblast maturation.

*SPP1* expression was increased after CD14-positive cell depletion. However, its product osteopontin should not be considered an exclusive marker of mature bone formation, as it also contributes to cell adhesion, matrix remodeling, immune signaling, and the regulation of mineral deposition [19]. Increased *SPP1* expression in the presence of reduced mineralization may therefore reflect an activated matrix-remodeling phenotype rather than successful terminal osteoblast maturation.

*BGLAP* expression and the proportion of osteocalcin-positive cells remained largely unchanged, providing no evidence for an increase in mature osteoblasts. Nevertheless, the reduced mineralization capacity indicates impaired functional maturation and suggests that CD14-positive cells support efficient matrix mineralization, potentially through macrophage-derived paracrine or contact-dependent signals. M2-like macrophages have been reported to promote osteogenic differentiation through soluble mediators, including TGF-β, VEGF and IGF-1 [13]. In addition, the close association of osteomacs with osteoblasts along bone-forming surfaces raises the possibility that direct cell contact supports osteoblast survival or late-stage maturation. Loss of these signals could therefore preserve or enrich early osteogenic features while impairing terminal matrix mineralization. However, these mechanisms were not directly examined in the present study and require validation using conditioned-medium, transwell, and direct coculture experiments.

Overall, the combined increase in CD271- and ALP-positive cells and *SPP1* expression, together with slightly reduced RUNX2 positivity, unchanged osteocalcin, and decreased mineralization, may indicate an uncoupling of early osteogenic features from terminal functional maturation. In bone, macrophage-lineage cells are not merely inflammatory contaminants, but can act as resident osteal macrophages. These cells are located in proximity to osteoblasts and have been shown to support osteoblast function and bone formation. Accordingly, depletion of CD14-positive cells may remove macrophage-derived signals required for late osteoblast maturation and mineral deposition.

In addition to changes in osteoblast-associated markers, depletion of CD14-positive cells resulted in the expected downregulation of monocyte/macrophage-associated markers, including *CD14*, *CD45*, *CD68*, *CD86*, *CD163*, and *CD209*. This finding confirms the effective reduction in the myeloid cell component within the osteoblast cultures. Conversely, the CD14-positive fraction showed a macrophage-related expression profile with increased levels of *CD68* and *CD163*, suggesting enrichment of macrophage-like cells with a predominantly anti-inflammatory, M2-like phenotype. This interpretation should, however, be made with caution, as macrophage polarization is highly plastic and cannot be fully defined by a limited marker set.

The clinical context of the present study is relevant in this regard. The osteoblast cultures were derived from patients undergoing total hip arthroplasty due to degenerative or metabolic hip disease, most commonly osteoarthritis or osteoporosis. Both conditions are increasingly recognized as being associated with immune-mediated alterations in the bone and joint microenvironment [3]. Although macrophage populations in osteoarthritis and osteoporosis are heterogeneous and cannot be reduced to a binary M1/M2 classification, macrophages expressing *CD68* and *CD163* are commonly associated with tissue-remodeling, reparative, and immunoregulatory functions. Thus, the predominance of *CD68*- and *CD163*-positive cells within the CD14-positive fraction suggests a macrophage population with an M2-like or osteomac-like phenotype rather than a predominantly pro-inflammatory M1-like state. This phenotype may reflect an adaptive response to chronic tissue damage and ongoing remodeling within the diseased bone and joint microenvironment from which the cells were obtained.

Importantly, this macrophage phenotype may also help explain the divergent osteoblast marker pattern observed after CD14-positive cell depletion. Rather than primarily removing a pro-inflammatory inhibitory compartment, CD14-positive cell depletion may eliminate macrophage-derived signals that regulate the balance between osteoblast progenitor maintenance, differentiation, and matrix formation. The reduction in mineral deposition suggests that *CD68*- and *CD163*-positive macrophages provide supportive signals required for later stages of osteoblast maturation and matrix mineralization. Such effects would be consistent with an osteomac-like function, as resident bone-associated macrophages have been implicated in osteoblast support, matrix formation, and bone homeostasis. Collectively, these findings indicate that the co-isolated CD14-positive cells may exert stage-specific and context-dependent effects on osteoblast differentiation, supporting late osteoblast function and mineralization while modulating early osteogenic marker expression. Their role should therefore not be interpreted within a simple pro- versus anti-osteogenic framework.

Future studies should therefore characterize the isolated CD14-positive cells in greater detail, including additional markers of monocyte/macrophage identity and polarization. Functional assays will also be required to determine whether these cells actively modulate osteoblast maturation, mineralization, and inflammatory signaling. In particular, it would be of interest to investigate whether M2-like macrophages support terminal osteoblast differentiation and mineralization more effectively than M1-like macrophages, whether induction of an M2-like phenotype within mixed osteoblast–macrophage cultures can restore or enhance OCN expression and matrix mineralization, whether osteomac-specific depletion is a central factor or whether the observed effects are independent of macrophage polarization.

Interestingly, although basal mineralization decreased after CD14-positive cell depletion, cytokine treatment increased mineralization irrespective of the presence or absence of CD14-positive cells. This observation is consistent with our previous findings in non-depleted human osteoblast cultures, in which cytokine stimulation enhanced mineralization capacity [15,16]. The current data extend these observations by showing that the pro-mineralizing effect of cytokine treatment is preserved even after depletion of CD14-positive cells.

This finding suggests that the mineralization response to cytokine stimulation is not primarily dependent on the presence of CD14-positive monocyte/macrophage-lineage cells. Instead, cytokines may directly modulate osteoblast activity and promote matrix mineralization through osteoblast-intrinsic signaling pathways. Alternatively, the remaining CD14-negative cell fraction may still contain stromal or immune-modulatory cell populations capable of responding to cytokines and indirectly supporting mineralization. However, because depletion of CD14-positive cells did not attenuate cytokine-induced mineralization, the CD14-positive compartment does not appear to be required for this effect under the present culture conditions.

This is particularly relevant in light of the marker-specific trends observed after CD14-positive cell depletion. Although some markers showed patterns that may be compatible with altered osteoblast maturation, these changes were donor-dependent and cannot be assigned confidently to a specific differentiation stage. The reduction in basal mineralization nevertheless indicates that osteogenic marker expression and functional matrix formation may be partially uncoupled in this culture system. The preserved cytokine-induced mineralization response further suggests that cytokine stimulation can overcome, at least in part, the reduced basal mineralization capacity of CD14-depleted cultures.

## 5. Conclusions

Together, these findings suggest that CD14-positive cells support basal osteoblast maturation and mineralization but are not essential mediators of the mineralization-promoting effect of cytokine treatment. Future studies should clarify whether cytokine-induced mineralization occurs through direct activation of osteoblast signaling pathways, through modulation of extracellular matrix composition or through effects on residual non-CD14-positive cell populations.

## Figures and Tables

**Table 1:** Primer list used for quantitative real-time PCR.

Target Gene	Forward Sequence (5′→3′)	Reverse Sequence (5′→3′)
*CD14*	CGGAAGACTTATCGACCATGGAGC	TGCAGACGCAGCGGAAATCT
*ALPL*	GCAGGCAGCTTGACCTCCTC	GCATGGGGGCCAGACCAAAG
*BGLAP*	CTCCTCGCCCTATTGGCCCT	CTGCTTGGACACAAAGGCTGC
*SPP1*	CACTCCAGTTGTCCCCACAGTAG	TCTGTAGCATCAGGGTACTGGATGT
*RUNX2*	AGTGGACGAGGCAAGAGTTT	TGTCTGTGCCTTCTGGGTTC
*NGFR*	CACCACCGACAACCTCATCCC	CTTGCAGCTGTTCCACCTCTTGA
*PTPRC*	ATCATCACCTAGCAGTTCATGCAGC	CAGCATGCGTCCTTTCTCCACT
*CD68*	CAGCTTTGGATTCATGCAGGACC	AGCCGAGAATGTCCACTGTGC
*CD86*	GGGGAGCCTGAGCCACAAAA	ATCAAGGCAGGGGCAGGGTA
*CD163*	AAAGCCACAACAGGTCGCTCA	AGCCGCTGTCTCTGTCTTCG
*CD209*	TAACCGCTTCACCTGGATGGGA	CCAATACTGCTTGAAGCTGGGCAA
*TFRC*	TTCAGGTCAAAGACAGCGCTCA	CTATACGCCACATAACCCCCAGG

**Table 2:** Antibody list used for immunofluorescence staining.

Antibody/Conjugate	Reference Number	Company
CD14 (UCH-M1) mouse anti-human	sc-1182	Santa Cruz Biotechnology (Dallas, TX, USA)
ALP (A-10) mouse anti-human	sc-271431	Santa Cruz Biotechnology (Dallas, TX, USA)
OCN rabbit anti-human	PA5-11849	Invitrogen (Waltham, MA, USA)
OPN (7C5H12) mouse anti-human	MA5-17180	Invitrogen (Waltham, MA, USA)
RUNX2 (M-70) rabbit anti-human	sc-10758	Santa Cruz Biotechnology (Dallas, TX, USA)
CD271 rabbit anti-human	bs-0161R	Bioss (Woburn, MA, USA)
CD45 (2B11) mouse anti-human	sc-20056	Santa Cruz Biotechnology (Dallas, TX, USA)
CD68 (KP1) mouse anti-human	sc-20060	Santa Cruz Biotechnology (Dallas, TX, USA)
CD86 rabbit anti-human	bs-1035R	Bioss (Woburn, MA, USA)
CD163 rabbit anti-human	bs-23127R	Bioss (Woburn, MA, USA)
DC SIGN/CD209 (UW60.1) mouse anti-human	AM26379PU-N	OriGene (Rockville, MD, USA)

## Data Availability

The raw data supporting the conclusions of the article will be made available by the authors on request.

## Supplementary Materials

The following supporting information can be downloaded at: https://www.mdpi.com/article/10.3390/biology15161420/s1. Table S1: Donor characteristics including age, sex, diagnosis, and medication (N/A = information not available). Table S2: Overview of the donor cell cultures obtained using the different isolation methods (hOB-I and hOB-II) and included in the analyses presented in Figure 1, Figure 2 and Figure 3. Figure S1: Distribution of osteogenic/stromal and monocyte/macrophage lineage marker expression in hOB-II and CD14-depleted hOB-II cultures. The immunofluorescence data presented as mean percentages in Figure 2B are shown here as low-to-high plots with median lines to illustrate the variability between individual biological donors (*n* = 3, 4 technical replicates). The percentages of cells positive for the markers were determined in hOB-II and CD14-depleted hOB-II cultures by immunofluorescence staining and quantified using Fiji/ImageJ.

## Abbreviations

The following abbreviations are used in this manuscript:

DMEM	Dulbecco’s Modified Eagle Medium
DMSO	Dimethyl Sulfoxide
GM	Growth Medium
MACS	Magnetic-Activated Cell Sorting
MTT	Dimethylthiazolyl blue tetrazolium bromide
OIM	Osteogenesis Induction Medium
TFRC	Transferrin receptor protein 1
CD	Cluster of differentiation
ALPL/ALP	Alkaline phosphatase
BGLAP/OCN	Bone gamma-carboxygluatamate protein/Osteocalcin
SPP1/OPN	Secreted phosphoprotein 1/Osteopontin
RUNX2	Runt-related transcription factor 2
NGFR	Nerve growth factor receptor
PTPRC	Protein tyrosine phosphatase receptor Type C
IL-1β	Interleukin 1 beta
IL-6	Interleukin 6
TNF-α	Tumor necrosis factor alpha
hOB	Primary human osteoblasts
