# Supplementary material for: CD14-Positive Cells Support Osteoblast Viability and Mineralization Without Altering Proinflammatory Cytokine Responsiveness
Source: Biology (Basel). 2026 Aug 18;15(16):1420. doi: 10.3390/biology15161420 (PMC13510259; doi:10.3390/biology15161420)
Supplement: Supplementary file 1 [file biology-15-01420-s001.zip › biology-4439697-supplementary.pdf]

Supplements

Supplementary Table S1: Donor characteristics including age, sex, diagnosis, and medication *(N/A = information not available)*.

donor	sex	age	diagnosis	medication
hOB-I_1	f	88	medial femoral neck fracture	rivaroxaban, buprenorphine patch, moxonidine, torsemide, spironolactone, ramipril, bisoprolol
hOB-I_2	f	70	*N/A*	*N/A*
hOB-I_3	m	74	osteoarthritis	eliquis, amiodarone, metoprolol, Aldactone, Torem, atorvastatin
hOB-I_4	f	87	osteoarthritis	amitriptyline, amlodipine, vitamin D3, levodopa, ramipril, quetiapine, pantoprazole
hOB-I_5	f	55	osteoarthritis	none
hOB-I_6	m	68	*N/A*	*N/A*
hOB-I_7	m	65	osteoarthritis	atorvastatin
hOB-I_8	m	75	osteoarthritis	edoxaban, levothyroxine
hOB-II_1	f	70	*N/A*	*N/A*
hOB-II_2	f	62	medial femoral neck fracture	none
hOB-II_3	m	61	osteoarthritis	allopurinol, ezetimibe, atorvastatin, candesartan, cinacalcet, heparin, lercanidipine
hOB-II_4	m	58	osteoarthritis	tirzepatide, tramadol, carvedilol, diclofenac, pantoprazole, spironolactone
hOB-II_5	m	70	medial femoral neck fracture	bisoprolol, amlodipine, valsartan, hydrochlorothiazide
hOB-II_6	f	90	medial femoral neck fracture	apixaban, bisoprolol, digoxin
hOB-II_7	m	83	medial femoral neck fracture	dapagliflozin, formoterol, pravastatin, vitamin D3, carvedilol, pregabalin, torsemide, ipratropium bromide, albuterol
hOB-II_8	f	65	osteoarthritis	omeprazole, prednisone, torsemide, spironolactone, vitamin D3, aspirin, ursodeoxycholic acid, ornithine aspartate, alendronate, cyanocobalamin

Supplementary Table S2: Overview of the donor cell cultures obtained using the different isolation methods (hOB-I and hOB-II) and included in the analyses presented in Figures 1-3.

**hOB-I donors**	**analysis**	
	**Fig. 1 (A, B)**	**Fig. 1 (C)**
hOB-I_1	✓	✓
hOB-I_2	✓	✓
hOB-I_3	✓	✓
hOB-I_4	✓	✓
hOB-I_5	✓	✓
hOB-I_6	✓	✓
hOB-I_7	✓	✓
hOB-I_8	✓	✓

**hOB-II donors**	**analysis**				
	**Fig. 1 (A, B)**	**Fig. 1 (C)**	**Fig. 2 (A)**	**Fig. 2 (B)**	**Fig. 3 (C)**
hOB-II_1	✓	✓	✓	✓	✓
hOB-II_2	✓	✓			✓
hOB-II_3	✓	✓	✓		✓
hOB-II_4	✓	✓	✓	✓	✓
hOB-II_5	✓	✓	✓		✓
hOB-II_6	✓	✓	✓	✓	✓
hOB-II_7	✓				
hOB-II_8	✓	✓	✓		✓

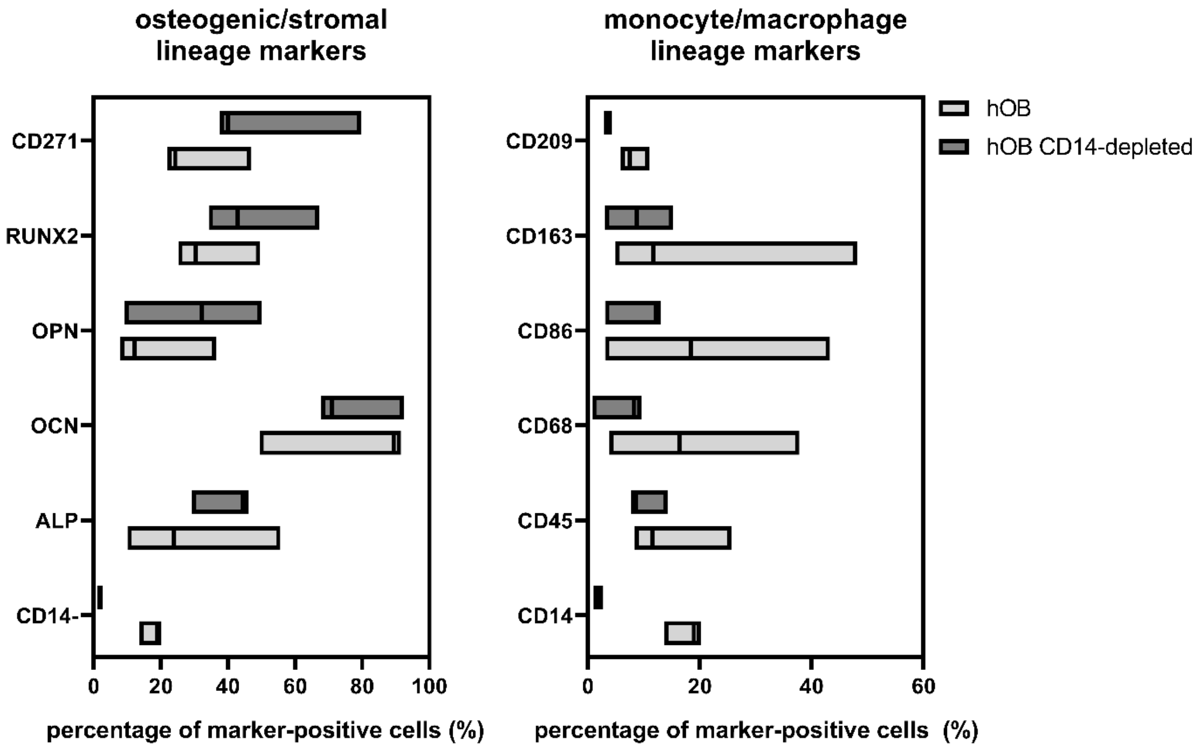

Supplementary Figure S1: Distribution of osteogenic/stromal and monocyte/macrophage lineage marker expression in hOB-II and CD14-depleted hOB-II cultures. The immunofluorescence data presented as mean percentages in Figure 2B are shown here as low-to-high plots with median lines to illustrate the variability between individual biological donors (n = 3, 4 technical replicates). The percentages of cells positive for the markers were determined in hOB-II and CD14-depleted hOB-II cultures by immunofluorescence staining and quantified using Fiji/ImageJ.
